# A microProtein repressor complex in the shoot meristem controls the transition to flowering

**DOI:** 10.1093/plphys/kiab235

**Published:** 2021-05-20

**Authors:** Vandasue L. Rodrigues, Ulla Dolde, Bin Sun, Anko Blaakmeer, Daniel Straub, Tenai Eguen, Esther Botterweg-Paredes, Shinyoung Hong, Moritz Graeff, Man-Wah Li, Joshua M. Gendron, Stephan Wenkel

**Affiliations:** 1 Department of Plant and Environmental Sciences, University of Copenhagen, Denmark; 2 Copenhagen Plant Science Centre, University of Copenhagen, Thorvaldsensvej 40, 1871 Frederiksberg C, Denmark; 3 Department of Molecular, Cellular and Developmental Biology, Yale University, New Haven CT 06511, USA; 4 NovoCrops Center, University of Copenhagen, Thorvaldsensvej 40, 1871 Frederiksberg C, Denmark

## Abstract

MicroProteins are potent post-translational regulators. In Arabidopsis (*Arabidopsis thaliana*), the miP1a/b microProteins delay floral transition by forming a complex with CONSTANS (CO) and the co-repressor protein TOPLESS. To better understand the function of the miP1a microProtein in floral repression, we performed a genetic suppressor screen to identify ***su****ppressors of* ***m****iP1a* (*sum*) *function*. One mutant, *sum1*, exhibited strong suppression of the miP1a-induced late-flowering phenotype. Mapping of *sum1* identified another allele of the gene encoding the histone H3K4 demethylase JUMONJI14 (JMJ14), which is required for miP1a function. Plants carrying mutations in *JMJ14* exhibit an early flowering phenotype that is largely dependent on *CO* activity, supporting an additional role for CO in the repressive complex. We further investigated whether miP1a function involves chromatin modification, performed whole-genome methylome sequencing studies with plants ectopically expressing *miP1a*, and identified differentially methylated regions (DMRs). Among these DMRs is the promoter of *FLOWERING LOCUS T* (*FT*), the prime target of miP1a that is ectopically methylated in a *JMJ14*-dependent manner. Moreover, when aberrantly expressed at the shoot apex, *CO* induces early flowering, but only when *JMJ14* is mutated. Detailed analysis of the genetic interaction among CO, JMJ14, miP1a/b, and TPL revealed a potential role for CO as a repressor of flowering in the shoot apical meristem (SAM). Altogether, our results suggest that a repressor complex operates in the SAM, likely to maintain it in an undifferentiated state until leaf-derived florigen signals induce SAM conversion into a floral meristem.

## Teaser

A mapping-by-sequencing approach allows identification of a suppressor of miP1a function, and the combination of proteomics and genomics reveals a repressor complex in the shoot meristem.

## Introduction

Annual plants, such as the model plant Arabidopsis (*Arabidopsis thaliana*), induce flowering only once during their lifetime, and this transition occurs in a nonreversible manner; once Arabidopsis commits to flowering, it cannot return to the vegetative growth state. In order to maximize reproductive success, plants integrate seasonal information, such as temperature and day-length, to initiate flowering only under the most optimal conditions. Since Arabidopsis is a long-day plant, it will start flowering when nights are short, thus restricting flowering to long-day periods such as summer.

The molecular network underpinning the photoperiodic flowering response has been elucidated using mutants and ecotypes that show variations in their respective flowering behavior (Imaizumi and Kay, 2006; Andres and Coupland, 2012). A central component of the photoperiodic flowering time pathway is the CONSTANS (CO) transcription factor (Putterill et al., 1995). Both *CO* mRNA and protein exhibit diurnal patterns of expression, but the protein can only accumulate at the end of long days (Suarez-Lopez et al., 2001; Valverde et al., 2004). Once CO protein is present, it acts as a transcriptional activator in leaves and induces expression of *FLOWERING LOCUS T* (*FT*; Samach et al., 2000). Both CO stability and its interaction with the *FT* promoter are mediated by a set of PSEUDO RESPONSE REGULATOR proteins (Hayama et al., 2017).

Arabidopsis leaves act as photoperiod sensors, and both *CO* and *FT* are expressed and active in the leaf vasculature. When expressed from a phloem-specific promoter, CO is able to fully rescue the late-flowering phenotype of *co* loss-of-function mutant plants; however, the expression of CO in the shoot apical meristem (SAM) does not complement the late-flowering phenotype (An et al., 2004). This contrasts with the finding that expression of *FT* in either the shoot meristem or the leaf vasculature is effective in triggering an early flowering response (An et al., 2004). Later, it was revealed that CO acts in the phloem to induce *FT* expression, and the resulting FT protein acts as a systemic florigen signal that travels from the leaves to the shoot meristem where it initiates the conversion of the vegetative leaf-producing meristem into a reproductive flower-producing meristem (Corbesier et al., 2007; Jaeger and Wigge, 2007; Mathieu et al., 2007; Tamaki et al., 2007).

Recently, we identified two Arabidopsis microProteins, miP1a and miP1b, that can interact with CO and, when overexpressed, cause a late flowering phenotype (Graeff et al., 2016). MicroProteins are small single-domain proteins that can exist as individual genes in the genomes of eukaryotes. MicroProteins are sequence-related to larger, multidomain proteins, and have evolved during genome evolution via amplification and subsequent partial degeneration. A hallmark of microProtein function is the presence of a single protein domain, often a protein–protein interaction domain, allowing the microProtein to exert dominant-negative modes of action by sequestering target proteins (Staudt and Wenkel, 2011; Eguen et al., 2015). In the case of miP1a/b, the mode of action is, however, not by a simple sequestration, but rather by the formation of a higher-order complex (Graeff et al., 2016). In this complex, the microProteins bridge between CO and the TOPLESS (TPL) co-repressor protein. To identify other components required for miP1a to repress flowering, we carried out an EMS mutagenesis screen for plants ectopically expressing *miP1a* that flower very late. In this EMS screen, we identified ***SU****PPRESSOR OF* ***M****IP1A-1* (*SUM1*), which has a frame-shift mutation in the gene encoding the Histone 3 Lysine 4 (H3K4)–demethylase JUMONJI14 (JMJ14). This mutation in *JMJ14* causes early flowering in *miP1a*-overexpressing plants. *JMJ14* has a known role in the regulation of flowering (Yang et al., 2010). H3K4 methylation is associated with active chromatin, hence removing H3K4 methylation may contribute to repression. Plants carrying loss-of-function mutations in *JMJ14* display an early flowering phenotype, express higher levels of *FT*, and have increased levels of H3K4 methylation in the *FT* promoter (Yang et al., 2010). JMJ14 also plays a role in RNA silencing and has been shown to influence DNA methylation in the process of silencing transposon transcripts (Searle et al., 2010).

This manuscript reports the identification of JMJ14 as a component required for the miP1a/b-repressor complex to suppress flowering. Because the role of JMJ14 in directing chromatin changes includes DNA-methylation, we performed whole-genome bisulfite sequencing with transgenic plants overexpressing *miP1a* and identified a region of differential methylation in the *FT* promoter. In order to identify more components of the potential repressor complex, we performed enrichment proteomics studies with miP1a, miP1b, TPL, and JMJ; these confirmed known interacting proteins and identified several other potential interactors. Mutations in *JMJ14* suppress the late-flowering phenotype exerted by ectopic expression of both miP1a and miP1b, but cannot complement late-flowering *co* mutants, indicating that JMJ14 function is dependent on CO. The finding that CO is unable to induce flowering when expressed in the SAM suggests that CO might have SAM-specific functions. Both miP1a and miP1b,TPL and JMJ14 are co-expressed with CO in the SAM where they could act to repress flowering. Exploring this hypothesis, we aberrantly expressed CO in the SAM of a *jmj14* mutant and observed a strong early flowering phenotype. Taken together, our findings indicate that the *FT* gene is actively repressed in the shoot apex by a repressor complex likely involving CO/CO-like transcription factors, microProteins miP1a/b, TPL, and JMJ14. This repressor complex prevents flowering until the leaf-derived FT protein triggers the transition to the reproductive growth phase.

## Results

### Isolation of *sum1*, a loss-of-function allele required for miP1a to suppress flowering

**Figure 1:**
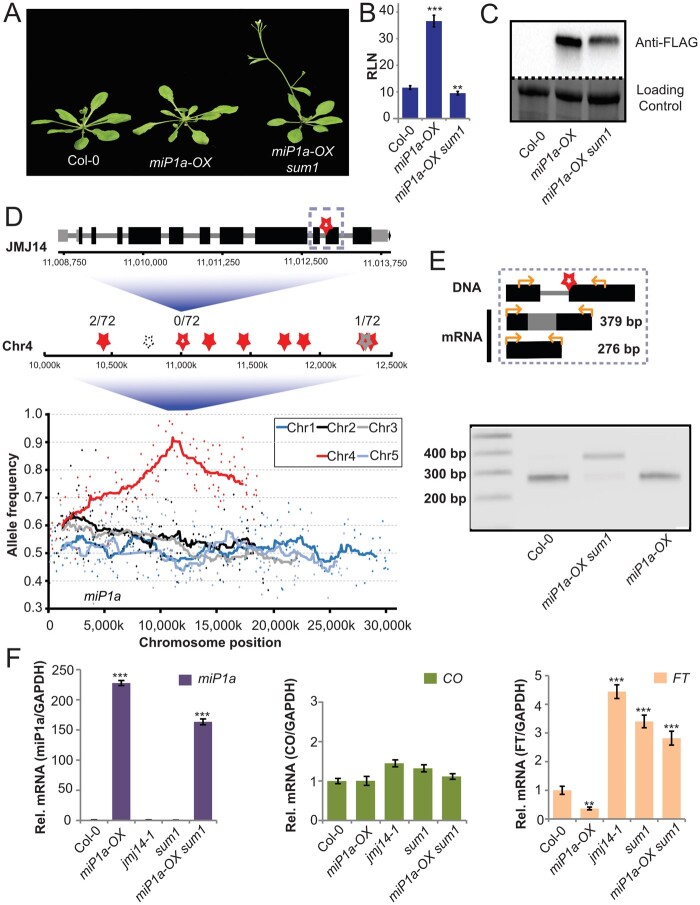
Mapping and molecular characterization of the *sum1* mutant phenotype. A, Phenotype of the M2 suppressor mutant *sum1* in the *miP1a-OX* background compared to the Col-0 WT and the *miP1a-OX* progenitor when grown in a long-day (LD) regime. Pictures of rosettes were digitally extracted for comparison. B, Determination of flowering time by counting the number of rosette leaves (RLN) at bolting in LD. Significant differences in leaf numbers between the *miP1a-OX sum1* line compared to *miP1a-OX* progenitor were observed. *N* =6–9 ±sd, **P <0.01, ***P <0.001. C, Analysis of protein levels of FLAG-tagged miP1a protein in the *miP1a-OX sum1* line compared to *miP1a-OX*. No signal was detected in the WT. Coomassie staining is shown as the loading control. D, Top: *JMJ14* gene model with G to A SNP (boxed) in the splice site before the second exon (black box: exon, gray: UTR, star: SNP). Middle: Stars indicate SNPs in a mapping interval of chromosome 4 (filled red: CDS; open red: splice site; gray: UTR; dashed: intergenic) with observed WT allele frequency numbers in F2 backcross generation (total 36 plants) identified by restriction fragment length polymorphism (RFLP) and/or amplification-refractory mutation system (ARMS). Bottom: Five Arabidopsis chromosomes with allele frequency of background corrected SNPs measured by mapping-by-sequencing. E, Top: model of the intron retained product (379 bp) and the correctly spliced product (276 bp mRNA). Orange arrows show position of primers, red star the position of the splice site mutation. Bottom: PCR amplification of the cDNA from Col-0 WT, *miP1a-OX sum1*, and *miP1a-OX* lines. *MiP1a-OX sum1* shows a predominantly higher product indicating intron retention due to a splicing defect. F, Quantification by RT-qPCR of *miP1a*, *CO*, and *FT* levels. *MiP1a* transgene levels are expressed at a high level in the *miP1a-OX sum1* line. While *CO* levels are not significantly different, there is a significant increase in the *FT* levels in the *miP1a-OX sum1* compared to *miP1a-OX*. Error bars depict the sd of four technical replicates, **P<0.005, ***P<0.001 determined by Student’s *t* test

MiP1a/b-type microProteins interact with CO through their B-Box domain and interact with TPL via a five amino-acid stretch at the carboxy-terminal end (Graeff et al., 2016)—the finding that TPL is required for the microProteins to exert their strong repressive potential points toward the existence of a higher-order repressor complex. To identify components of such a repressor, we performed an EMS suppressor mutagenesis with plants ectopically expressing FLAG-miP1a. In total, we identified 25 potential suppressor mutants of which 4 mutants no longer expressed the transgene, 13 showed expression levels between the wild-type (WT) and the *FLAG-miP1a* overexpressor, and 8 plants showed expression levels comparable to the parental plants (Supplemental Figure S1). One suppressor, named *sum1*, was isolated and studied in detail. Under long-day conditions, Col-0 WT plants flower rapidly, producing only a small number of rosette leaves, in contrast to transgenic plants over-expressing FLAG-miP1a (Figure 1, A and B), which are late flowering and produce many leaves before transitioning to flowering. Plants that are homozygous for the *sum1* mutation flower slightly earlier than the WT plants, despite the presence of the *FLAG-miP1a* transgene.

High levels of transgene expression do not always correspond with a high translation rate. In order to determine the protein expression level of miP1a, we measured the levels of FLAG-miP1a protein in the parental transgenic plant and the *sum1* background. We detect slightly lower levels of FLAG-miP1a in the *sum1* mutant compared to the WT, but the protein is still highly abundant (Figure 1C). These findings indicate that the factor encoded by *SUM1* is required for the miP1a microProtein to repress flowering.

### The microProtein repressor complex requires JMJ14 activity

We aimed to identify the causal mutation underlying the *sum1* phenotype. Therefore, we isolated DNA from 20 *sum1* suppressor mutants that came out of a segregating F2 population from a back-cross to Col-0. All 20 individuals showed tolerance to the herbicide BASTA, had high levels of *miP1a* mRNA and exhibited an early flowering phenotype. Whole-genome sequencing of this pool of suppressor mutants and the respective parental plant identified 591 EMS-induced single nucleotide polymorphisms (SNPs) with a strong frequency enrichment in the middle of chromosome 4 (Supplementary Data Set 1). At the summit region of the enrichment peak, we identified a mutation affecting a splice junction in the *JMJ14* gene (Figure 1D). Further characterization of an additional 36 segregating suppressor mutants revealed that all 72 examined chromosomes carried the *jmj14* mutation while flanking mutations were still segregating (Figure 1D). Next, we tested if the identified *jmj14* mutation would interfere with the correct splicing of the *JMJ14* transcript. Reverse transcription polymerase chain reaction amplifications spanning the intron in question using cDNAs prepared from Col-0, transgenic *miP1a-OX*, and *miP1a-OX sum1* plants revealed intron-retention in the *sum1* background (Figure 1E). The retained intron results in a premature stop-codon and the resultant mutated JMJ14 protein lacks the carboxy-terminal FY-rich (FRYC) domain that might engage in protein–protein interactions (Pless et al., 2011). To confirm that *sum1* is indeed the causal mutation that suppresses miP1a function, we crossed homozygous *miP1a-OX sum1* plants with either homozygous *jmj14-1* or *jmj14-3* mutant plants. Both cases indicated the resultant nullizygous offspring had an early flowering phenotype (Supplemental Figure S2), which confirmed that *JMJ14* is the causal gene and encodes the protein likely required for the floral repression imposed by ectopic miP1a expression. Additional gene expression profiling experiments revealed that *miP1a* mRNA levels were highly upregulated in transgenic *miP1a-OX* and *miP1a-OX sum1* plants. *CO* mRNA levels were slightly upregulated in *jmj14-1* and *sum-1* mutant plants while *FT* mRNA was highly abundant in *jmj14-1*, *sum-1*, and *miP1a-OX sum1* plants, explaining the early flowering behavior (Figure 1F).

In summary, our results show that *FT* is under constant repression by a JMJ14-containing silencing complex. Plants lacking *JMJ14* show a slightly early flowering behavior in long-day conditions that can be attributed to the de-repression of *FT*. The observation that ectopic microProtein expression is unable to repress *FT* in a *jmj14* mutant background suggests either that miP1a and JMJ14 act together as part of a repressor complex or that miP1a acts upstream of the JMJ14-induced floral repression pathway. A recent study suggests that mutations in *JMJ14* can result in a re-activation of genomic regions that have undergone post-transcriptional gene silencing and additionally, can decrease the expression of transgenes by affecting the chromatin of the transgene (Le Masson et al., 2012). However, this is not the case in our study because *miP1a-OX sum1* (i.e. *miP1a-OX jmj14*) plants flowered earlier than the WT and did not show an intermediate flowering phenotype. In addition, *miP1a-OX sum1* transgenic plants were fully tolerant to the herbicide BASTA and the double-heterozygote from the back-cross to the WT exhibited a very late flowering phenotype.

### JMJ14 controls flowering in a CO-dependent manner

**Figure 2:**
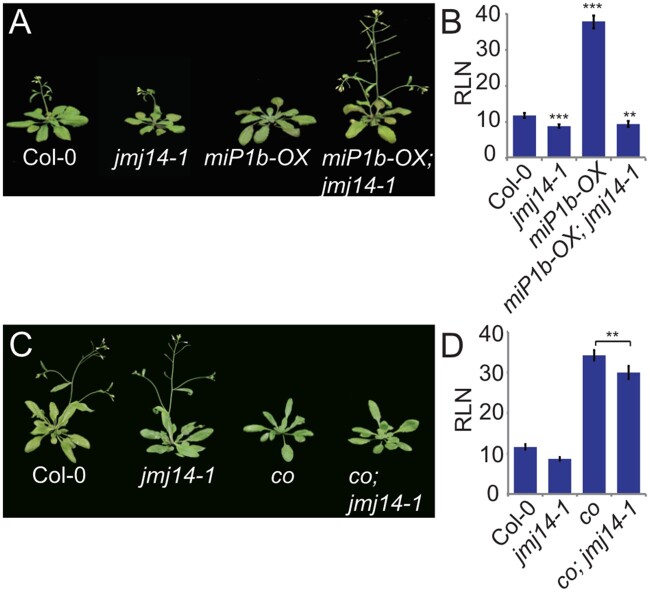
Genetic analysis of crosses between *jmj14* and late flowering mutants grown in inductive LD conditions. A, Flowering phenotype of *jmj14-1* mutants crossed with transgenic *miP1b-OX* plants grown in LD conditions. Pictures of plants were digitally extracted for comparison. B, Determination of flowering time by counting the number of rosette leaves (RLN) at the bolting stage of the WT, *jmj14-1, miP1b-OX*, and *miP1b-OX jmj14-1* plants. *N* = 4–10, error bars show the sd, **P <0.005, ***P <0.001 determined by Student’s *t* test. C, Phenotype of *jmj14-1* introduced into *co* background is extremely late flowering compared to the parent *jmj14-1* and Col-0 WT. Pictures of plants were digitally extracted for comparison. D, Determination of flowering time by counting the number of rosette leaves at the bolting stage of the WT, *jmj14-1*, *co*, and *co jmj14-1* mutant plants. *N* = 4–10, error bars show the sd, **P <0.005, ***P <0.001 determined by Student’s *t* test

As shown before, the loss of JMJ14 function disabled the ability of miP1a to repress flowering (Figure 1A). The miP1b microProtein is closely related to miP1a and can also strongly delay flowering when expressed at high levels (Graeff et al., 2016). To test if the observed suppressor phenotype is specific to miP1a, we crossed late-flowering *miP1b-OX* plants into the *jmj14-1* mutant. Transgenic *miP1b-OX* plants that were homozygous for *jmj14-1* also showed an early flowering phenotype, indicating that both miP1a and miP1b need JMJ14 to execute their repressive potential (Figure 2, A and B). However, when we crossed *jmj14-1* into a *co* null mutant (*co-SAIL*), we did not observe complementation of the late-flowering phenotype of *co*, and respective *co jmj14-1* double mutants flowered only slightly earlier than *co* single mutants (Figure 2, C and D). This suggests that JMJ14 may be required for the post-translational inhibition of CO function or its integration into a repressor complex. Loss of JMJ14 function attenuates the repressor complex, hence plants flower early. The finding that mutations in *jmj14* do not fully complement the late-flowering phenotype of *co* mutants implies that JMJ14 does not operate independently of CO. In agreement with this, we found no strong promotion of flowering in *jmj14* mutants when grown in short days, a condition where CO is not active (Supplemental Figure S3).

### The miP1a microProtein interacts with the promoter of *FT*, which coincides with the deposition of repressive chromatin marks

**Figure 3:**
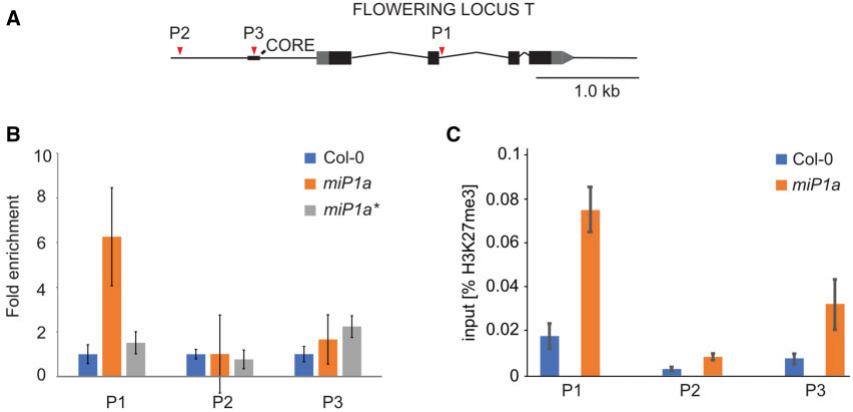
ChIP reveals direct association of miP1a with *FT* chromatin and deposition of H3K27me3 repressive chromatin marks. A, Schematic drawing of the *FT* genomic locus. P1–P3 depict positions tested by ChIP (red arrowheads); CORE, CO response element; gray box/arrow 5′- and 3′-UTR. B, ChIP experiment with the WT and transgenic plants expressing either FLAG-miP1a or FLAG-miP1a*, the latter carrying mutations in the B-Box domain that prevent an interaction with CONSTANS. Gene model in (A) depicts the positions amplified by qPCR (P1–P3). Diagram shows the fold enrichment of four technical replicates ±sd relative to Col-0 WT plants. C, ChIP experiment using anti-H3K27me3 antibodies. Enrichment of four technical replicates ±sd is plotted relative to the input control samples

The finding that miP1a/b physically interacts with CO, the TPL co-repressor, and potentially with JMJ14 in a higher-order complex suggests that this complex might associate with chromatin to exert its function. To confirm that miP1a is part of a DNA-binding complex, we performed chromatin-immunoprecipitation (ChIP) experiments with transgenic plants expressing either FLAG-miP1a or FLAG-miP1a*. The latter is a miP1a B-Box-dead variant in which all cysteine and histidine residues of the B-Box zinc finger had been changed to alanine. As a consequence, the mutant miP1a* protein can no longer interact with CO, but retains its ability to interact with TPL (Graeff et al., 2016). CO acts as transcriptional activator of *FT* and has been shown to directly and physically interact with the *FT* promoter (Tiwari et al., 2010; Song et al., 2012; Hayama et al., 2017). We used primers amplifying the area around the previously identified CO-response element (CORE; P3; Tiwari et al., 2010), plus additional primer pairs amplifying up-stream (P2) and downstream (P1) of the CORE (Figure 3A). In these experiments, we did not detect enrichment that would support binding of miP1a around the CORE region but instead detected enrichment indicative of miP1a binding at the second exon/intron boundary of the *FT* gene (Figure 3B). No enrichment was observed with transgenic plants expressing FLAG-miP1a*, indicating that a functional B-Box is required, most likely to associate with a DNA-binding B-Box-containing protein that mediates the chromatin-interaction. Interestingly, FLAG-miP1a binding occurred near a recently identified CO-binding site (Hayama et al., 2017), supporting the idea that it could be CO that is part of the miP1a DNA-binding complex.

To gain insight into the mechanism through which miP1-type microProteins regulate *FT* expression, we analyzed whether the binding of miP1a to the *FT* chromatin is associated with the deposition of repressive chromatin marks. We tested the trimethylation of lysine 27 of histone H3 (H3K27me3) and in line with the binding of miP1a to the *FT* chromatin, we found the highest enrichment of H3K27me3 at the P1 position where we also observed the strongest binding of miP1a (Figure 3C). Low H3K27me3 levels were observed at the very distant position (P2), and modest enrichment at the proximal promoter close to the CO-response element (P3). In summary, these results demonstrate that a miP1a-containing microProtein complex physically interacts with the *FT* chromatin, which is accompanied with the deposition of repressive histone marks. These histone marks might then serve as a signal for additional DNA methylation and the formation of heterochromatin.

### Ectopic miP1a expression causes methylation changes in the *FT* promoter

**Figure 4:**
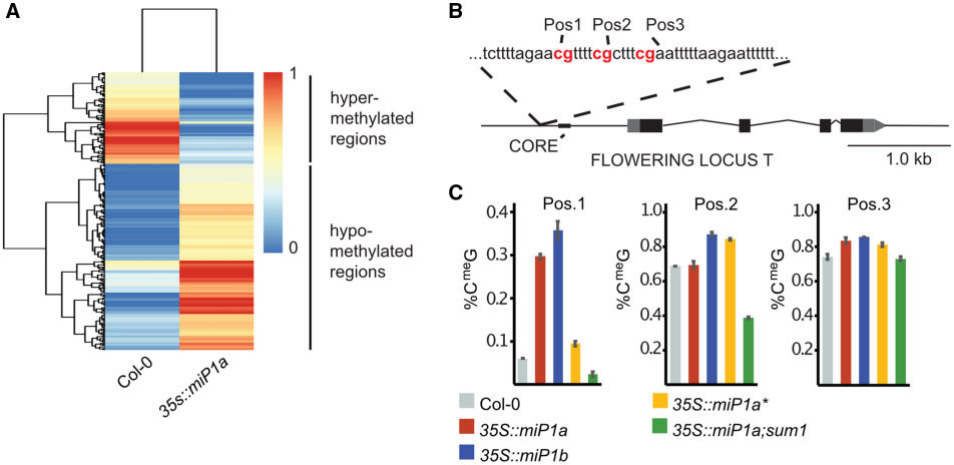
Whole-genome bisulfite sequencing reveals differential methylation in transgenic plants overexpressing *miP1a*. A, Identification of DMRs in Col-0 versus the *35S::miPa1* transgenic plants using whole-genome bisulfite sequencing. B, Overview of the *FT* promoter. *CORE*, *CONSTANS RESPONSE ELEMENT*; CGs in red (positions 1–3); gray box/arrow represent the 5′- and 3′-UTRs. C, Bisulfite amplicon sequencing analysis. Depicted are the three CG positions in the DMR and the percent methylation detected at each site; *N* = 5,000 ±sd

Having established that miP1a/b engage with CO and TPL to bind DNA and alter the chromatin landscape, we extended these studies to the investigation of DNA methylation. Transcriptional repression is often accompanied by changes in the chromatin, and JMJ14 has been shown to strongly affect DNA methylation (Searle et al., 2010). We decided to pursue a whole-genome methylome analysis to obtain an unbiased picture of the role of miP1a in the repression of flowering. Bisulfite-sequencing was performed with transgenic plants overexpressing *miP1a* (*35S::FLAG-miP1a*) and with Col-0, which served as a reference. In total, we found 270 differentially methylated regions (DMRs) that had a methylation difference above 40% (Figure 4A;  Supplementary Data Set 2). There were clear differences in the general methylation patterns, with two-thirds of the regions in *miP1a-OX* being hypermethylated compared to the Col-0 WT.

To further determine whether loci showing altered methylation states also show differential gene expression levels in plants ectopically expressing *miP1a*, we compared the methylome of *miP1a-OX* plants with differentially expressed genes in *miP1a-OX* that were previously obtained by RNA-seq (Graeff et al., 2016). This analysis revealed no strong connection between methylation state and expression levels globally. However, we did find that the master circadian clock regulator *CIRCADIAN CLOCK ASSOCIATED 1* (*CCA1*) was hypomethylated and transcriptionally upregulated in *miP1a-OX* plants compared to the WT (Table 1). Conversely, *FT* exhibited increased methylation and very low expression levels in *miP1a-OX* compared to the WT plants (Table 1). Looking at the regions upstream of the *FT* promoter, we identified three CpG sites (Figure 4B). Our whole-genome bisulfite sequencing experiment resulted in a coverage depth of around 20-fold, and many of the identified differential methylations could be a result of experimental noise. In order to further enrich for reads at the three positions in the *FT* promoter and to check the methylation status of other mutants in this region, we performed a targeted bisulfite sequencing experiment with a 5,000-fold coverage. We specifically amplified the region containing the three differentially methylated cytosines in Col-0, *35S::miP1a*, *35S::miP1b*, 35S::miP1a*, and *35S::miP1a;sum1* lines. Sequencing results indicated that the most substantial difference was in position 1, where Col-0 showed 6% methylation, compared to 29% and 35% methylation in *35S::miP1a* and *35S::miP1b*, respectively (Figure 4C). 35S::miP1a*, the B-Box dead version of miP1a, showed a methylation level closer to Col 0 at 9%. Interestingly, at 2%, *35S::miP1a;sum1* showed methylation amounts even lower than those of Col 0. At position 2, we detected a strong reduction in the methylation amount in *35S::miP1a;sum1* plants compared to Col-0. The third position showed no strong changes. Taken together, these findings demonstrate that influencing DNA methylation is part of the function of miP1a. This is supported by the finding that *sum1 (jmj14)*, a suppressor of miP1a function, flowers early despite high *miP1a* mRNA levels and reverses the DNA methylation changes observed in the promoter of *FT*.

**Table 1:** Loci showing differential methylation and differential gene expression in transgenic *miP1a-OX* plants versus WT

AGI code	Annotation	[%] me WT	[%] me miP1a-OX	[FC]	FDR
A/hypomethylated and downregulated genes in miP1a-OX					
AT4G29030	Putative membrane lipoprotein	67	0	0.0319	1.02E-13
AT4G14390	Ankyrin repeat family protein	60	0	0.1021	1.67E-33
AT2G35380	Peroxidase superfamily protein	58	0	0.0090	5.86E-04
AT1G62510	Bifunctional inhibitor/lipid-transfer protein	56	0	0.0576	2.55E-15
AT3G20865	AGP40	48	0	0.1755	9.67E-04
B/hypermethylated and downregulated genes in miP1a-OX					
AT4G20000	VQ motif-containing protein	0	71	0.1062	1.31E-08
AT1G66830	Leucine-rich repeat protein kinase family protein	0	67	0.2013	4.70E-06
AT3G22231	PCC1 | PATHOGEN AND CIRCADIAN CONTROLLED 1	0	42	0.2451	1.94E-28
AT2G25130	ARM repeat superfamily protein	3	61	0.0060	1.10E-05
AT1G12940	ATNRT2.5, NRT2.5 | nitrate transporter2.5	20	68	0.0070	5.89E-05
AT2G35980	NHL10, ATNHL10, YLS9	12	57	0.1620	4.24E-06
AT1G65480	FT	25	67	0.0004	5.70E-58
AT4G12500	Bifunctional inhibitor/lipid-transfer protein	39	81	0.0058	6.44E-06
AT5G40010	AATP1, AAA-ATPase 1	21	63	0.1510	7.30E-04
C/hypomethylated and upregulated genes in miP1a-OX					
AT2G37260	ATWRKY44, DSL1, TTG2	68	0	5.7	4.23E-20
AT2G39330	JAL23, jacalin-related lectin 23	60	0	11.6	2.40E-50
AT1G03940	HXXXD-type acyl-transferase family protein	53	0	4.1	4.28E-12
AT4G21830	ATMSRB7, MSRB7, methionine sulfoxide reductase B7	49	0	114.1	4.10E-04
AT5G26260	TRAF-like family protein	46	0	15.3	3.50E-06
AT2G46830	CCA1, circadian clock associated 1	45	0	4.1	1.44E-12
AT4G14090	UDP-Glycosyltransferase superfamily protein	66	23	8.8	6.03E-56
AT1G71030	ATMYBL2, MYBL2, MYB-like 2	57	16	4.6	2.82E-15
D/hypermethylated and upregulated genes in miP1a-OX					
AT2G37770	NAD(P)-linked oxidoreductase superfamily protein	11	52	11.1	4.55E-18
AT5G41315	GL3, GL3, MYC6.2, basic helix-loop-helix	40	83	7.6	4.66E-22
AT1G04220	KCS2, 3-ketoacyl-CoA synthase 2	0	46	5.6	1.52E-31
AT1G52000	Mannose-binding lectin superfamily protein	12	59	12.5	1.18E-79
AT3G25180	CYP82G1, cytochrome P450, family	9	59	51.7	4.89E-13
AT4G23680	Polyketide cyclase/dehydrase	25	75	20.2	1.78E-08
AT1G06620	2-oxoglutarate (2OG) and Fe(II)-dependent oxygenase	5	56	4.5	8.35E-15
AT1G22240	APUM8, PUM8, pumilio 8	22	73	218.7	1.85E-07
AT3G50770	CML41, calmodulin-like 41	38	91	6.0	5.57E-44
AT1G34180	anac016, NAC016, NAC domain containing protein 16	24	80	5.5	2.64E-18
AT1G52030	F-ATMBP, MBP1.2, MBP2, myrosinase-binding protein 2	0	58	21.6	1.94E-37
AT2G07732	Ribulose bisphosphate carboxylase	0	60	4.1	3.61E-26
AT3G10320	Glycosyltransferase family 61 protein	15	77	19.7	1.45E-11
AT3G24982	ATRLP40, RLP40, receptor-like protein 40	31	100	6.9	1.77E-26

FC, fold change in mRNA-seq data set; FDR, false discovery rate.

### Dissection of the microProtein repressor complex by mass spectrometry

**Figure 5:**
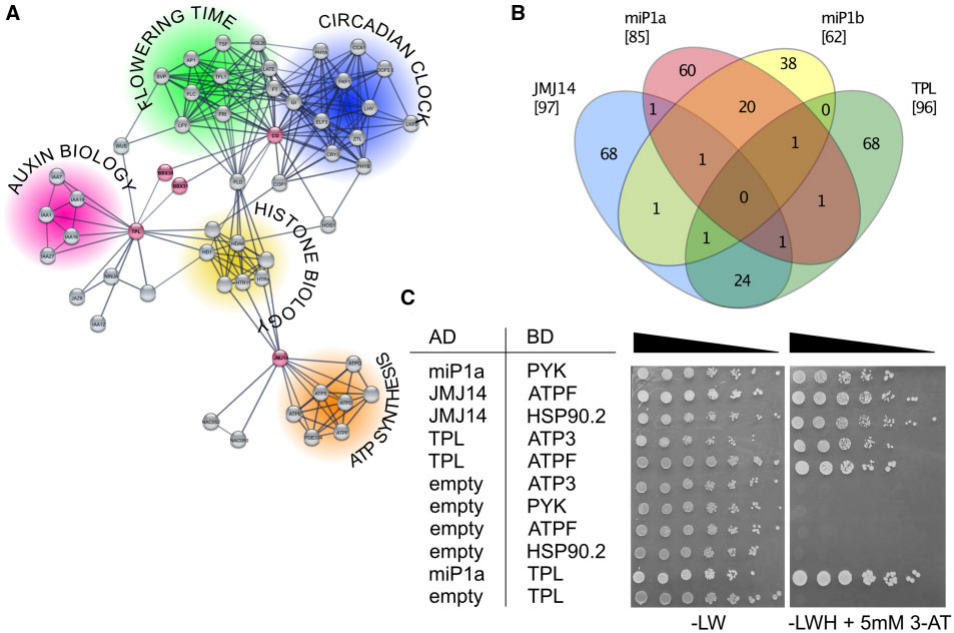
Comparative enrichment proteomic analysis of miP1a-, miP1b-, TPL-, and JMJ14-interacting proteins. A, Modified STRING network depicting high confidence (0.700) connections of TPL, CO, miP1A, miP1B, and JMJ14. CO is connected to flowering time and circadian clock networks, TPL is connected to an auxin network, and JMJ14 to ATP-synthesis. The miP1a/b microProteins connect TPL to CO and a cluster of histone/histone-related proteins connects TPL and JMJ14. TPL, CO, JMJ14, miP1a, and miP1b in pink; putative interactors in gray. B, Venn diagram depicting the number of proteins co-purified with FLAG-miP1a, FLAG-miP1b, FLAG-JMJ14, and FLAG-TPL. Nonspecific interactors identified in experiments with either WT plants or plants expressing FLAG-GFP have been subtracted. C, Yeast-two-hybrid interactions were tested by transformations of empty vector or of fusions of miP1a, JMJ14, and TPL to the Gal4 activation domain (AD), and fusions of potential interactors to the Gal4 binding domain (BD). Shown are the growth of serial dilutions of co-transformants on nonselective (-LW) and selective (-LWH) SD medium. The latter medium was supplemented with 5 mM of the competitive HIS-inhibitor 3-aminotriazole (3-AT)

Having established that miP1a interacts with CO and TPL to repress flowering, and that this repression seems to involve additional players such as JMJ14, we sought to identify additional partners involved in the microProtein complex. Using the STRING database (https://string-db.org), we extracted all high confidence connections between miP1a, miP1b, CO, TPL, and JMJ14. This network analysis revealed no direct connection between TPL and JMJ14, but an indirect connection via proteins involved in histone biology. In addition, we found that JMJ14 is connected to a range of proteins involved in the synthesis of ATP (Figure 5A).

To experimentally identify proteins involved in the miP1-repressor complex, we performed affinity-purification mass-spectrometry with transgenic plants overexpressing FLAG-miP1a and FLAG-miP1b (Supplemental Data Set 3). As control for false-positive interactors, we also performed immunoprecipitations (IPs) with nontransgenic WT plants and plants overexpressing FLAG-GFP protein. Proteins that were identified in two or more replicates but not found in either WT or FLAG-GFP IP were considered high confidence interactors. We identified 85 proteins interacting with miP1a and 62 proteins interacting with miP1b. In total, 20 proteins were in common between miP1a and miP1b. These include, among others, the CO-like 4 (COL4) protein, CO-like 9 (COL9), and TPL (Table 2). This confirmed that the miP1a/b microProteins interact with B-Box transcription factors and associate with TPL-like co-repressor proteins *in vivo*. However, we did not identify CO in these pull-down experiments, which is likely a result of the low abundance of the CO protein. Alternatively, miP1a/b might form different types of repressor complexes that also involve other CO-like proteins. In order to find additional proteins interacting with either TPL or JMJ14, which might shed light on the formation of a potential higher-order repressor complex, we also generated plants overexpressing FLAG-TPL and FLAG-JMJ14 to co-purify additional interacting proteins (Supplementary Data Set 4). Similar to miP1a/b, we also performed parallel IPs with Col-0 and transgenic plants expressing FLAG-GFP, but this time performed an additional active coal purification step prior to injection into the mass spectrometer. Comparative analysis of these four data sets revealed 97 JMJ14-interacting proteins and 96 TPL-interacting proteins (Figure 5B). In total, we identified 24 proteins co-precipitating with JMJ14 and TPL. The JMJ14 data set included a protein group of NAC transcription factors NAC50 and NAC52 that had previously been found to interact with JMJ14 (Ning et al., 2015). TPL co-precipitated all other TPL-related (TPR) proteins, supporting the recent finding that TPL/TPR proteins contain a tetramerization interface (Martin-Arevalillo et al., 2017). These examples confirm that our mass spectrometry–immunoprecipitation (MS–IP) strategy identified *bona fide* JMJ14- and TPL-interacting proteins. We note that we could not detect previously identified TPL/TPR-interacting repression-domain containing transcription factors (Causier et al., 2012). This could indicate that these interactions are either transient or that they are stabilized by additional interacting proteins that were not present in our conditions. In addition, we did not find a single protein that interacted with miP1a/b, TPL, and JMJ14 that would support the formation of a higher-order repressor complex.

**Table 2:** Interacting proteins identified by enrichment proteomics

Accession number	Description	Category	mip1a	mip1b	TPL	JMJ14
	**Bait**					
At3g21890	B-box-type zinc finger family protein; miP1a	Bait	X	X^a^		
At4g15248	B-box-type zinc finger family protein; miP1b	Bait		X		
At1g15750	TPL, WSIP1, Transducin family protein/WD-40 repeat family protein	Bait	X^a^	X^a^	X	
At4g20400	JMJ14, PKDM7B, JUMONJI 14	Bait				X
	**B-BOX proteins**					
At5g24930	ATCOL4, COL4, CO-like 4	Prey	X	X		
At3g07650	COL9, CO-like 9	Prey	X^a^	X		
At1g68190	B-box zinc finger family protein, BBX27	Prey	X	X^a^		
	**TOPLESS-related**					
At1g80490	TPR1, TOPLESS-related 1	Prey			X	
At3g16830	TPR2, TOPLESS-related 2	Prey	X^a^	X^a^	X	
At5g27030	TPR3, TOPLESS-related 3	Prey			X	
At3g15880	TPR4, WSIP2, WUS-interacting protein 2	Prey			X	
	**Flowering-related**					
At2g21060	ATCSP4, ATGRP2B, GRP2B, glycine-rich protein 2B	Prey			X	
At3g07050	GTP-binding family protein	Prey			X	
At3g22231	PCC1, pathogen and circadian controlled 1	Prey	X	X^a^		
At4g27890	HSP20-like chaperones superfamily protein	Prey		X		
At4g39100	SHL1, PHD finger family protein/bromo-adjacent homology	Prey			X^a^	X
At5g14530	Transducin/WD40 repeat-like superfamily protein	Prey			X^a^	X
	**Sugar-related**					
At1g35580	CINV1, cytosolic invertase 1	Prey		X^a^	X	
At5g20830	ASUS1, atsus1, SUS1, sucrose synthase 1	Prey	X^a^		X	X
	**Miscellaneous**					
At1g08420	BSL2, BRI1 suppressor 1 (BSU1)-like 2	Prey		X^a^		X
At1g13870	DRL1, calmodulin binding; purine nucleotide binding	Prey		X	X^a^	X^a^
At1g75600	Histone superfamily protein	Prey	X^a^		X	X^a^
At1g78370	ATGSTU20, GSTU20, glutathione S-transferase TAU 20	Prey			X	X
At3g10480	ANAC050, NAC050, NAC domain containing protein 50	Prey				X^a^
At3g10490	ANAC051, ANAC052, NAC052, NAC domain containing protein 52	Prey				X^a^
At3g50500	SNRK2-2, SNRK2.2, SPK-2-2, SRK2D, SNF1-related protein kinase 2.2	Prey			X	X
At5g59910	HTB4, Histone superfamily protein	Prey			X	X
At5g65430	14-3-3KAPPA, GF14 KAPPA, GRF8, general regulatory factor 8	Prey			X	X^a^

^a^ Identified in only one replicate.

To experimentally validate that some of the interactions we observed here would also occur in a different system, we performed directed yeast-two-hybrid experiments with candidate proteins identified by STRING or MS–IP. Here, we found that PYK (AT3G06610), which was identified by MS–IP to interact with both TPL and JMJ14, interacted with miP1a but not with either JMJ14 or TPL. Conversely, we observed an interaction among ATPF (ATCG00130), TPL, and JMJ14 in yeast, but ATPF interacted in MS–IPs with both miP1a and miP1b. We also detected an interaction between HSP90.2 and JMJ14, and used the interaction between miP1a and TPL as a positive control (Figure 5C). These results suggest that a higher-order protein complex comprising miP1-type microProteins and TPL and JMJ14 might exist, and the interaction could either be mediated through PYK or ATPF. Failure to detect interactions observed by MS–IP in yeast could indicate that the in planta complex contains interaction partners that stabilize the interaction and which are missing in yeast.

### Misexpression of *CO* in the shoot meristem accelerates flowering in *jmj14* mutant plants

**Figure 6:**
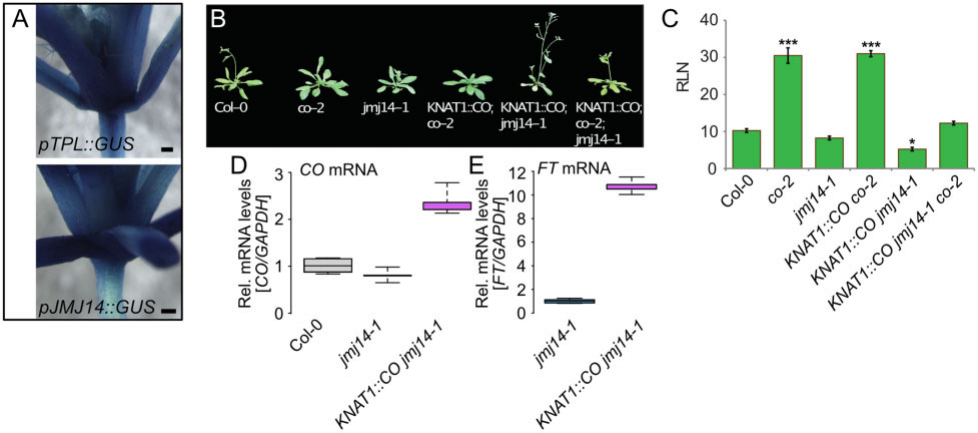
Expression of *CO* in the meristem of *jmj14* mutants rescues the late flowering phenotype of *co* mutants. A, Expression patterns of *TPL* (top) and *JMJ14* (bottom) determined by GUS-staining of *pTPL::GUS* and *pJMJ14::GUS* transgenic plants. Strong GUS expression was detected throughout the shoot apex; bar = 1 mm. B, Representative picture of plants. Pictures of plants were digitally extracted for comparison. C, Determination of flowering time by counting the number of rosette leaves (RLN) at the bolting stage of the WT, *co-2*, *jmj14-1*, *KNAT1::CO co-2, KNAT1::CO jmj14-1*, and *KNAT1::CO co-2 jmj14-1* mutant plants. *N* = 5 ±sd, *P <0.05, ***P <0.001 determined by Student’s *t* test. D, RT-qPCR using RNAs extracted from dissected SAMs from the WT (Col-0), *jmj14-1* and *KNAT1::CO jmj14-1* plants. E, RT-qPCRs using RNAs shown in (C). Plotted are *FT* mRNA levels relative to the *jmj14-1* mutant. In Col-0 WT plants, *FT* mRNA was below the level of detection. Shown is one biological replicate (D and E) of two that yielded similar results with five technical repeats. The center line of the box plots depicts the median and box limits indicate the 25th and 75th percentiles. The whiskers extend 1.5 times the interquartile range from the 25th and 75th percentiles

Measuring day length and the subsequent production of the florigenic signal(s) occurs in the leaves. Both *CO* and *FT* are expressed and active in the leaf vasculature (An et al., 2004). Surprisingly, *CO* is also expressed in the SAM where *FT* is absent (An et al., 2004; Graeff et al., 2016). This could indicate an activator-independent role of CO in the SAM. When expressed from the SAM-specific *KNAT1* promoter, CO was unable to rescue the late-flowering phenotype of *co* mutant plants (An et al., 2004). This contrasted findings with *FT*, where expression from the *KNAT1* promoter caused very early flowering, even in the late flowering *co* mutant background (An et al., 2004). We noted that besides *CO*, *miP1a* and *miP1b* (Graeff et al., 2016) showed robust expression in the SAM. To investigate the spatial expression pattern of *TPL* and *JMJ14* in the SAM, we obtained respective promoter-GUS reporter constructs that were recently published (Cattaneo et al., 2019; Kuhn et al., 2020). JMJ14 and TPL showed very strong, ubiquitous GUS expression in the SAM and leaves, supporting the notion that these factors are present in the SAM (Figure 6A). To assess if a potential JMJ14-containing repressor complex would operate in the SAM, we crossed *KNAT1::CO co-2* plants with *jmj14-1* mutant plants. When grown under inductive long-day conditions, we found that WT plants flowered early compared to *co-2* and *KNAT1::CO co-2* plants, confirming earlier findings that expression of CO in the SAM is not sufficient to induce flowering. However, we detected a very early flowering response when we introduced the *KNAT1::CO* transgene into the *jmj14* mutant background (Figure 6, B and C). Also in combination with a mutation in *co*, *KNAT1::CO jmj14 co-2* mutant plants flowered very early, supporting the idea that CO and JMJ14 are part of a repressor complex that acts in the SAM to repress *FT* expression.

To independently determine that CO can induce *FT* expression in the shoot meristem when JMJ14 is not active or present, we manually dissected shoot apices from Col-0 WT, *jmj14-1*, and *KNAT1::CO jmj14-1* plants to determine abundances of *CO* and *FT* mRNAs. This analysis revealed that the levels of *CO* mRNA were comparable between Col-0 and *jmj14-1* but increased in *KNAT1::CO jmj14-1* (Figure 6D). This finding confirms that *KNAT1::CO jmj14-1* plants indeed exhibit ectopically elevated levels of *CO* in the SAM, and that the early flowering phenotype of *jmj14-1* single mutant plants is not a result of ectopic *CO* expression in the meristem. When the expression of *FT* was analyzed in the same samples, we could not detect any *FT* mRNA in the meristem of the WT plants. This is consistent with previous findings that had shown expression of *CO* but not *FT* in the SAM (An et al., 2004; Tsutsui and Higashiyama, 2017). Because we were unable to detect *FT* in the meristem of WT plants, we normalized the data to the *jmj14-1* mutant in which we had detected robust levels of *FT* mRNA (Figure 6E). In *KNAT1::CO jmj14-1* plants we detected even higher levels of *FT*, explaining the early flowering phenotype of these plants (Figure 6, B, C, and E). In summary, these findings confirm that JMJ14 acts as an inhibitor of flowering in the shoot meristem. Meristematic expression of CO cannot induce early flowering. In the absence of JMJ14, CO is, however, able to induce *FT* in the meristem, indicating that a meristem-specific repressor complex exists that maintains the SAM in a nondifferentiated state until the leaf-derived florigen signal induces its conversion into a floral meristem.

To investigate the contribution of miP1a and miP1b in controlling flowering, we induced genomic deletions in WT plants using a CRISPR/Cas9-based approach (Tsutsui and Higashiyama, 2017). Both mutants that were generated had lost large fractions of respective coding sequences and no transcripts were detectable, suggesting that the mutants we created are true null mutants (Supplementary Figure S4). Both single mutants resembled WT plants with regard to their vegetative growth and flowering behavior, and we constructed *miP1a miP1b* double mutant plants by crossing. Analysis of the flowering behavior of this double mutant in long- and short-day conditions revealed no significant change compared to WT control plants (Supplementary Figure S5).

## Discussion

Different flowering time pathways operate in parallel to ensure that the transition to flowering occurs at the most optimal time point. CO regulates the floral transition in response to day length. Our previous work identified miP1a and miP1b as two microProteins able to repress flowering. Here we showed that miP1a can influence the methylation state of DNA, likely by participating in chromatin-modifying complexes. It remains unclear how many of the identified DMRs are biologically relevant for miP1a function. But, our study identified *FT* as one of the methylation targets in plants overexpressing miP1a. The effect of ectopic *FT* promoter methylation was confirmed by exhaustive amplicon deep-sequencing and because transgenic plants overexpressing *miP1a* and *miP1b* showed strong increases in DNA-methylation (Figure 4). In the case of *miP1a*, the observed increases in DNA-methylation were reversed in the *jmj14 (sum1)* mutant background. Because many methylation changes occur in a tissue-specific manner, it is conceivable that stronger differences could be detected by extracting tissue only from the meristem region. The fact that we observe genome-wide changes in the methylation status of transgenic *35S::miP1a* plants indicates, however, that one of the functions of miP1-type microProteins could be to recruit chromatin-modifying proteins through interaction with CO/CO-like transcription factors. Whether and to what extent the methylation of a single cytosine in the *FT* promoter is relevant for flowering time control is currently unclear. However, the effect was observed in independent biological replicates and by both whole-genome bisulfite sequencing and by amplicon bisulfite sequencing, and therefore, is unlikely to be an artifact. Moreover, it is well established that methylation of a single cytosine strongly influences the binding of the human ETS protein to DNA (Gaston and Fried, 1995).

Our studies also provide further evidence that miP1a/b-type microProteins associate with DNA-binding complexes. Using a modified ChIP strategy, we could show that miP1a interacts with the *FT* locus (Figure 3). Interestingly, we found that the region to which the miP1a complex bound was different from the region where we observed ectopic DNA methylation. Previous studies have, however, revealed looping of the *FT* chromatin, which brings distant regions close to the proximal promoter (Cao et al., 2014). These loops could be stabilized by a NUCLEAR FACTOR Y/CO complex and it seems plausible that the microProtein–repressor complex partially associates with these structures to initiate chromatin changes. We find that the miP1a microProtein has the potential to strongly affect the level of *FT* expression. Methylation of individual cytosines in promoter regions can influence the overall transcription status of genes by preventing transcription factor binding (Medvedeva et al., 2014). Thus, it seems possible that the changes we observed antagonize activation of *FT*.

**Figure 7:**
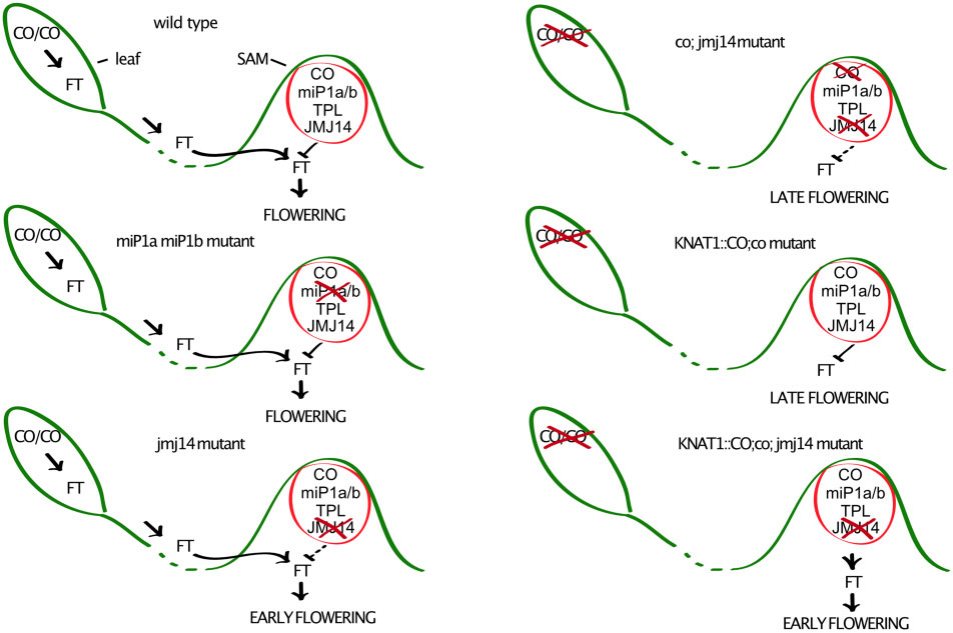
Hypothetical model of the CO-miP1-TPL-JMJ14 genetic interactions in LD conditions. In WT plants, CO upregulates *FT* expression in leaves in response to LDs. FT protein travels to the SAM where it induces flowering. In the SAM, CO-miP1-TPL, together with JMJ14, act to repress *FT* expression, allowing flowering to occur exclusively when the leaf-derived FT reaches the SAM. The concomitant removal of *miP1a* and *miP1b* does not affect the repressor complex. In *jmj14* mutants, the repressive activity in the SAM is reduced, resulting in early flowering. The *co; jmj14* double mutant plant flowers late because no leaf-derived FT is reaching the SAM. The expression of CO in the meristem (*KNAT1::CO;co* mutant) does not rescue the late flowering phenotype of *co* mutants. The ectopic expression of *KNAT1::CO* in *jmj14 co* double mutant plants causes early flowering that is likely caused by ectopic expression of *FT* in the SAM

In a complementary parallel approach, we found that mutations in the *JMJ14/SUM1* gene suppress miP1a function (Figure 1, A and B). JMJ14 is a histone demethylase, and it has been shown that the demethylation of histones results in subsequent DNA methylation, which was identified using bisulfite-sequencing (Greenberg et al., 2013). Thus, it seems that JMJ14 could be either part of the miP1a-repressor complex or at least be connected to it. Enrichment proteomic studies with miP1a, miP1b, TPL, and JMJ14 did not identify a common denominator able to bridge among all four proteins, but TPL and JMJ14 share ∼25% of the interactors. Thus, it appears that TPL and JMJ14 may function together as partners in different protein complexes, likely including the miP1-repressive complex. Support for this hypothesis comes from the genetic analysis of transgenic plants ectopically expressing *miP1a* or *miP1b* at high levels but which flower early when JMJ14 is absent. In WT plants, the florigenic signal (FT protein) is produced in the leaf and travels to the shoot to induce the conversion into a floral meristem (Figure 7). To prevent precocious flowering, we suggest that a repressor complex might act in the SAM in connection with the JMJ14 histone-demethylase to repress *FT*. In combination with a mutation in the *CO* gene, *jmj14-1 co* double mutants flowered late under inductive long-day conditions, indicating that the early flowering observed in *jmj14* single mutant plants depended on the activity of CO. Hence, *co jmj14* double mutants flowered late because no florigenic signals were coming from the leaves to the meristem, which is where the *jmj14* mutation affected the repressor complex (Figure 7). However, ectopic expression of CO in the SAM in *co jmj14* double mutants caused early flowering, likely because of the nonfunctional SAM-repressor complex, allowing CO to ectopically induce *FT* expression in the SAM (Figure 7). It is intriguing to speculate why the concerted loss of *miP1a* and *miP1b* did not result in stronger flowering time changes. The most logical explanation is genetic redundancy. Not only are miP1a/b are able to “recruit” CO into a complex that delays flowering but also the BBX19 protein has been shown to act in a similar fashion (Wang et al., 2014). Moreover, CO itself produces an alternative splice product that is able to antagonize the full-length product at the protein level (Gil et al., 2017). Thus, it seems likely that these factors, as well as other unknown factors, engage the flowering activator CO into a TPL/JMJ14-containing repressor.

Depending on the age of the plant, the environmental conditions or the tissue, specific transcription factors have been identified that can regulate the transition to flowering. Chromatin-modifying complexes containing polycomb group proteins and diverse histone-modifying enzymes fine-tune the chromatin state of the floral integrator gene *FT* in a plug-and-play fashion (Gu et al., 2013; Forderer et al., 2016; Wang et al., 2014). Here, we provide evidence that microProteins engage in repressor complexes that act to modify the chromatin of *FT*. These repressor complexes likely contain additional components, some of which might be found in the enrichment proteomics data sets we provide here (Table 2). The finding that mutations in *CO* lead to late flowering in the absence of *JMJ14* supports a role for CO in this repressive complex. Elucidating these control circuits in a spatiotemporal fashion will be the next steps in understanding how the balance of activating and repressing complexes triggers developmental transitions.

## Methods

### Plant material and growth conditions

Transgenic plants overexpressing miP1a, miP1b, and miP1a* are described in Graeff et al. (2016). The *jmj14-1* mutant corresponds to SALK_135712. For flowering time experiments, seeds were stratified 48 h at 4°C and grown on soil in a plant growth chamber under long-day light conditions (16-h light/8-h dark) at 22°C day/18°C night, or short-day light conditions (8-h light/16-h dark) at 22°C day/18°C night. Flowering time was measured by counting the number of rosette leaves at onset of bolting. Data are expressed as mean ± sd.

### EMS mutagenesis and growth of Arabidopsis

A seed stock of ∼1 mL homozygous transgenic *35S::FLAG-miP1a* seeds were immersed in 0.025% ethylmethanesulfonate (Sigma) overnight with gentle agitation. These M1 seeds were grown, self-pollinated, pooled and harvested. Approximately 1,000 M2 seeds from each original M1 pool were grown in soil under long-day conditions to identify early flowering suppressors of miP1a. Suppressors were categorized on the basis of leaf count at flowering. This was defined as plants that flowered with less than or an equal number of leaves at flowering as Col-0, which meant that they flowered substantially earlier when compared to the flowering time of the nonmutagenized parental transgenic plants. They were further characterized by quantification of the *miP1a* mRNA levels by reverse transcription quantitative polymerase chain reaction (RT-qPCR) and protein levels by western blot.

### Identification of mutants and construction of a mapping population

The early flowering *sum1* suppressor plant was backcrossed to the nonmutagenized Col-0 and the late flowering F1 offspring was allowed to self-pollinate. A population of F2 individuals was grown to identify segregating mutants. From 20 early flowering plants, one leaf disk of each plant was extracted by a leaf punch and pooled. For the control genome sequencing, five leaf discs each of four miP1a-OX plants were pooled separately. Genomic DNA of these two samples was extracted (DNeasy plant mini kit, QIAGEN). Novogene (Hongkong) prepared libraries and performed sequencing on an Illumina HiSeq4000 (350-bp insert size, 100-bp paired-end, 7 Gb data).

### Mapping-by-sequencing

More than 95% sequenced reads were mapped by Bowtie2 (v2.1.0; Langmead and Salzberg, 2012) using the TAIR9 genome assembly and TAIR10 annotation from Phytozome v10.3 (phytozome.org). SNP calling was performed using samtools and BCFtools (v0.1.19; Li et al., 2009). 1121 (Chr1: 288, Chr2: 233, Chr3: 235, Chr4: 164, Chr5: 201) background corrected EMS-induced SNP markers were identified by SHOREmap v3.2 (Schneeberger et al., 2009) using standard settings. Finally, 591 high-quality mutations (quality ≥100, reads supporting the predicted base ≥20) indicated a mapping interval of 2,500 kb on chromosome 4 that contained 10 mutations. The trend line is the average of all SNP allele frequencies in a sliding window (size: 2,500 kb; step: 100 kb).

### Gene expression analysis

RNA was extracted from a pool of 12 2-week-old plants from all lines under investigation for gene expression analysis using the Spectrum Plant Total RNA Kit (Sigma-Aldrich). RT-qPCR for miP1a, CO and FT was performed as described previously (Graeff et al., 2016).

### Whole-genome bisulfite sequencing

Genomic DNA was extracted from 12-d-old seedlings grown under LD conditions on MS plates (plant midi kit, QIAGEN), and BGI tech solutions (Hong Kong) prepared bisulfite treated libraries and performed sequencing on a Illumina HiSeq instrument (250–300 bp insert size, 150-bp paired-end, 5 Gb data per sample). Mapping was performed with BSseeker2 (v2.1.0; Guo et al., 2013) using Bowtie2 (v2.1.0; Langmead and Salzberg, 2012). TAIR9 genome assembly and TAIR10 annotation from Phytozome v10.3 (phytozome.org) were used. Genome coverage was calculated with bedtools (v2.17.0; Quinlan and Hall, 2010). Methylation levels were calculated as #C/(#C+#T) using Methpipe (v3.4.3). DMRs were defined by dividing the genome into 100-bp bins using bedtools (v2.17.0; Quinlan and Hall, 2010). For each bin, the number of methylated and unmethylated Cs was compared in mutant and WT using Fisher’s exact test (*P*≤0.01) and a minimum absolute methylation difference of 0.4. Heat maps of DMRs were generated by “pheatmap” package (v1.0.8) in R software (v3.2.2; R Development Core Team, 2011), and clusters were grouped by the complete linkage method with Euclidean distance measurement.

### Amplicon bisulfite sequencing

DNA extraction was performed according to manufacturer’s protocol using the (DNeasy plant mini kit, QIAGEN), followed by bisulfite treatment according to the online protocol Bisulphite Sequencing of Plant Genomic DNA (Aichinger and Kohler, 2010). Primers used in the amplification of the *FT* promoter target region were P1: GTATAATTATAAGAAAAGGTTGTTT; P2: TTAATAACCACTAATTTTTAATTTA. Libraries were constructed with Nextera XT DNA Library Preparation Kit and Nextera XT Index Kit (Illumina), sequenced on Illuminas MiSeq (v3 chemistry, PE 300 bp), adapter trimmed and demultiplexed to fastq by bcl2fastq2 (v2.19.1, Illumina). Half a million to one million reads were obtained per sample. Forward and reverse reads were merged with PEAR (v0.9.10; Zhang et al., 2014) and annealed by BSseeker2 (v2.1.0) (Guo et al., 2013) using Bowtie2 (v2.1.0; Langmead and Salzberg, 2012) to the genome sequence of the amplicon with around 90% success. BSseeker2 analyzes a maximum of 8,000 reads per genome position, therefore three subsets of around 5,000 reads were randomly chosen with samtools (v0.1.19; Li et al., 2009) format and these subsets were analyzed for their methylation level by BSseeker2.

### Protein purification for MS

Plant tissue from 3- to 4-week-old WT, GFP-FLAG-OX, miP1A-OX, and miP1B-OX Arabidopsis plants grown under LD conditions was harvested at the end of the long day and flash frozen in liquid nitrogen. The tissue was homogenized and resuspended in SII buffer (100-mM sodium phosphate, pH 8.0, 150-mM NaCl, 5-mM EDTA, 5-mM EGTA, 0.1% TX-100, protease inhibitor (cOmplete^TM^, EDTA-free Protease Inhibitor Cocktail), 1-mM phenylmethylsulfonyl fluoride (PMSF) and 1× phosphatase inhibitors), sonicated and clarified by centrifugation. The protein extract was bound to anti-FLAG M2 magnetic beads (Sigma-Aldrich) for 1 h. Protein bound beads were washed with SII buffer sans inhibitors, followed by washes with 25-mM ammonium bicarbonate buffer. The beads were flash frozen with liquid nitrogen prior to downstream analysis.

### MS parameters

Sample preparation: Proteins bound to anti-FLAG beads were subjected to on-bead digestion as follows: beads were washed three times with 10-mM ammonium bicarbonate (pH 7.5–8.0), trypsin was added to each sample, and digestion was performed overnight at 37°C. The supernatant was collected and dried by speed vac. The peptides were dissolved in 5% Formic Acid/0.1% trifluoroacetic acid (TFA), and protein concentration was determined by nanodrop measurement (A260/A280; Thermo Scientific Nanodrop 2000 UV-Vis Spectrophotometer). An amount of 0.5 μg (5 μL) of 0.1% TFA diluted protein extract was injected per sample for liquid chromatography with tandem MS (LC–MS/MS) analysis. LC–MS/MS analysis was performed on a Thermo Scientific Orbitrap Elite mass spectrometer equipped with a Waters nanoAcquity UPLC system utilizing a binary solvent system (Buffer A: 100% water, 0.1% formic acid; Buffer B: 100% acetonitrile, 0.1% formic acid). Trapping was performed at 5 μL·min^-1^, 97% Buffer A for 3 min using a Waters Symmetry C18 180 μm × 20 mm trap column. Peptides were separated using an ACQUITY UPLC PST (BEH) C18 nanoACQUITY Column 1.7 μm, 75 μm × 250 mm (37°C), and eluted at 300 nL·min^-1^ with the following gradient: 3% buffer B at initial conditions; 5% B at 3 min; 35% B at 140 min; 50% B at 155 min; 85% B at 160–165 min; return to initial conditions at 166 min. MS was acquired in the Orbitrap in profile mode over the 300–1,700 *m/z* range using 1 microscan, 30,000 resolution, AGC target of 1E6, and a full max ion time of 50 ms. Up to 15 MS/MS were collected per MS scan using collision-induced dissociation on species with an intensity threshold of 5,000 and charge states 2 and above. Data-dependent MS/MS were acquired in centroid mode in the ion trap using 1 microscan, AGC target of 2E4, full max IT of 100 ms, 2.0 *m/z* isolation window, and normalized collision energy of 35. Dynamic exclusion was enabled with a repeat count of 1, repeat duration of 30 s, exclusion list size of 500, and exclusion duration of 60 s.

### Protein identification database searching

All MS/MS spectra were searched using the Mascot algorithm (version 2.4.0) for uninterpreted MS/MS spectra after using the Mascot Distiller program to generate Mascot compatible files. The data were searched against the Swiss Protein database with taxonomy restricted to *A. thaliana*, and allowing for methionine oxidation as a variable modification. Peptide mass tolerance was set to 10 ppm and MS/MS fragment tolerance to 0.5 Da. Normal and decoy database searches were run to determine the false discovery rates, and the confidence level was set to 95% within the MASCOT search engine for protein hits based on randomness.

### Accession numbers

Sequence data from this article can be found in the NCBI Gene Expression Omnibus data libraries under accession numbers GSE173190, GSE173191, and GSE173192.

## Supplemental data

The following materials are available in the online version of this article.

**Supplemental Data Set S1.** Identification of differentially methylated regions in miP1a-OX versus Col-0 WT plants.

**Supplementary Data Set S2.** List of SNPs present in *miP1a-OX sum1* mutant plants, identified by whole genome sequencing.

**Supplementary Data Set S3.** Identification of miP1a and miP1b interacting proteins in comparison to proteins immunoprecipitated from WT and *35S::FLAG-GFP* transgenic plants.

**Supplementary Data Set S4.** Identification of TPL and JMJ14 interacting proteins in comparison to proteins immunoprecipitated from WT and *35S::FLAG-GFP* transgenic plants.

**Supplementary Figure S1.** Expression levels of the *miP1a* transgene in potential suppressor mutants.

**Supplementary Figure S2.** The *sum1* mutation is the phenotype-causing mutation.

**Supplementary Figure S3.** Flowering time analysis in short days.

**Supplementary Figure S4.** CRISPR/Cas9 mediated targeted gene knockout of *miP1a* and *miP1b*.

**Supplementary Figure S5.** Flowering time analysis of *miP1a miP1b* mutants in different photoperiods.

## Supplementary Material

kiab235_Supplementary_Data

## Notes

These authors contributed equally to this work (V.L.R., U.D., B.S.).

V.L.R., U.D., E.B.P., B.S., S.H., A.B., and M.G. performed the experiments; D.S. analyzed Illumina sequencing data; T.E., M.W.L., and J.G. performed and analyzed proteomics experiments; S.W. conceived the research and wrote the manuscript with input from all co-authors.

The author responsible for distribution of materials integral to the findings presented in this article in accordance with the policy described in the Instructions for Authors (https://academic.oup.com/plphys/pages/general-instructions) is: Stephan Wenkel (wenkel@plen.ku.dk).
