# Supplementary material for: A microProtein repressor complex in the shoot meristem controls the transition to flowering
Source: Plant Physiol. 2021 May 20;187(1):187–202. doi: 10.1093/plphys/kiab235 (PMC8418433; doi:10.1093/plphys/kiab235)
Supplement: kiab235_Supplementary_Data [file kiab235_supplementary_data.zip › pp.00466.2021-s01.pdf]

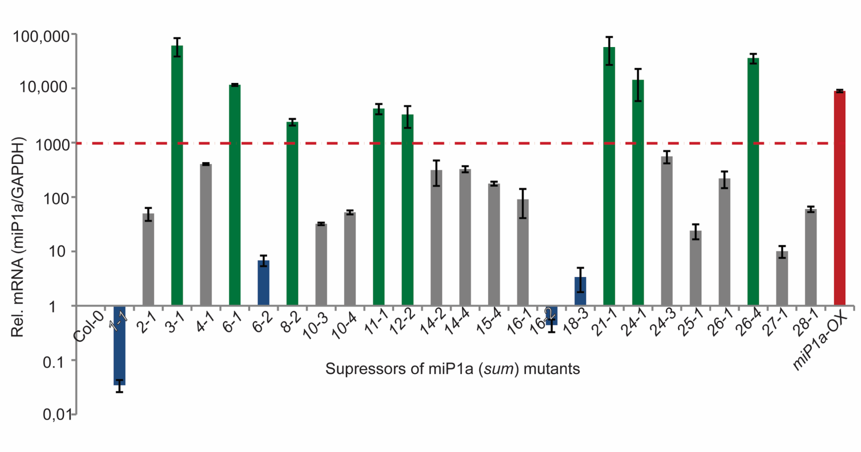

Supplementary Figure S1. Expression levels of the *miP1a* transgene in potential suppressor mutants. Individual plants showing high *miP1a* transcript levels (green bars), comparable to the FLAG-miP1a parental overexpression line (red bar) were isolated for further analysis. Lines with intermediate expression levels (gray bars) and low expression levels (blue bars) were discarded. Error bars show the SD of four technical replicates.

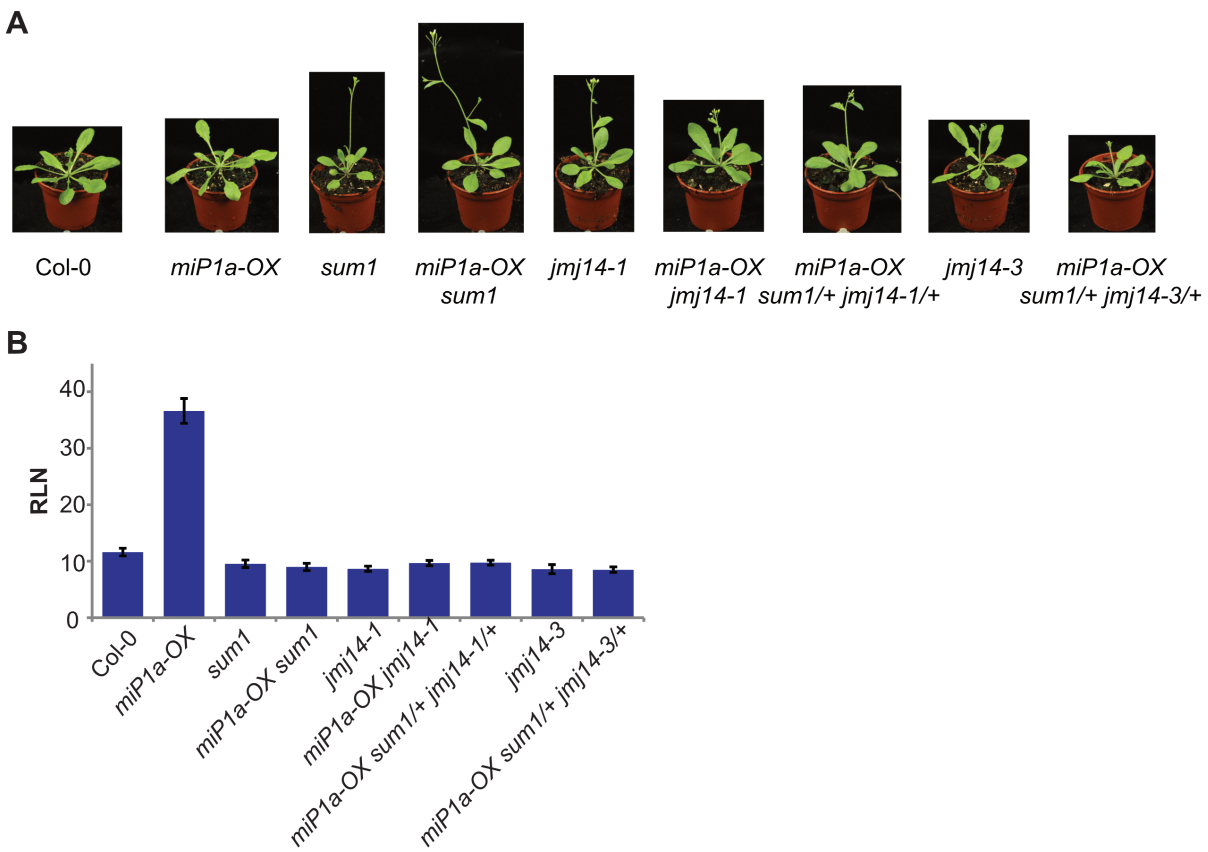

Supplementary Figure S2. The *sum1* mutation is the phenotype-causing mutation. (A) A characterized *JMJ14* mutant plant *jmj14-1* was crossed with a late flowering *miP1a-OX* plant. The resultant double homozygous offspring, *miP1a-OX jmj14-1,* is early flowering. Additionally, the isolated mutant line *miP1a-OX sum1* was crossed into mutants *jmj14-1* and *jmj14-3*. Resultant F1 cross also show an early flowering phenotype. Images of Col-0, *miP1a-OX* and *miP1a-OK sum1* are the same as shown in Figure 1A. (B) Flowering time counts of the genetic crosses +/- SD. RLN: Number of rosette leaves at the transition to flowering.

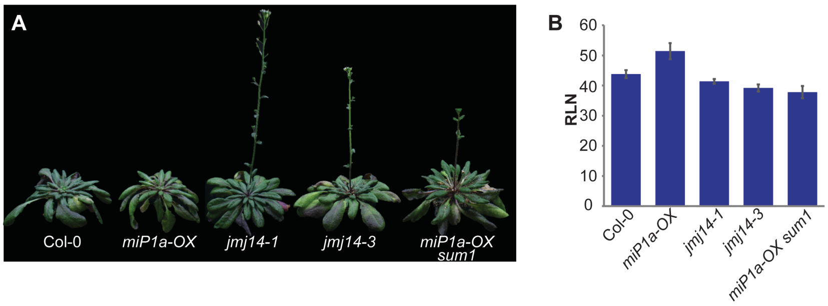

Supplementary Figure S3. Flowering time analysis in short days. (A) Flowering time of *jmj14* mutants under short day conditions. (8h light / 16h dark). Pictures of plants were digitally extracted for comparison. (B) Flowering time analysis by counting the number of rosette leaves (RLN) of the genetic crosses +/- SD.

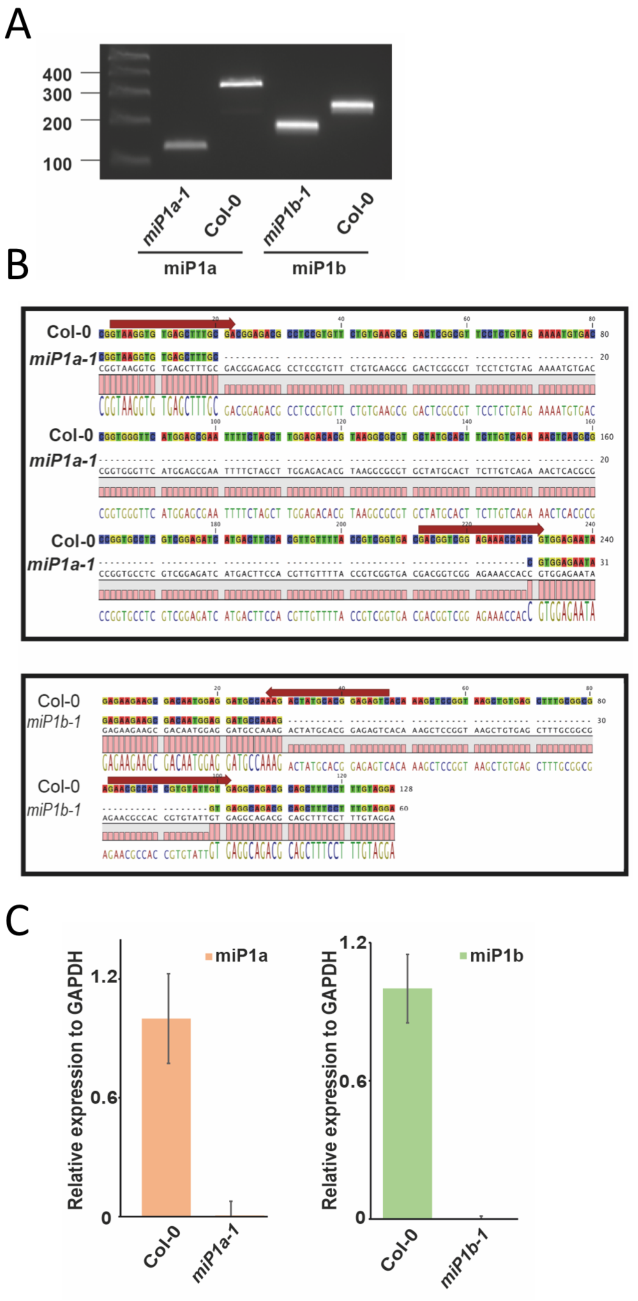

Supplementary Figure S4. CRISPR /Cas9 mediated targeted gene knockout of miP1a and miP1b.

(A) PCR testing the gene truncations using primers flanking the sgRNA target sites. *miP1a-1* (lane 1) is approximately 210bp smaller than wildtype miP1a PCR product (lane 2), and *miP1b-1* (lane 3) is approximately 70bp smaller than wildtype miP1b PCR product (lane 4). Size differences correspond to the distances between the respective sgRNA pairs.

(B) Nucleotide alignments showing the location of the sgRNA target sequences for *miP1a* (top) and *miP1b* (bottom) respectively. Red arrows indicate the sgRNAs.

(C) qRT-PCR determining the transcript levels of endogenous *miP1a* and *miP1b* in *miP1a-1* and *miP1b-1* mutants compared to the Col-0 wildtype. No transcripts were detectable in *miP1a- 1* and *miP1b-1*. Error bars indicate the standard deviation of four technical replicates.

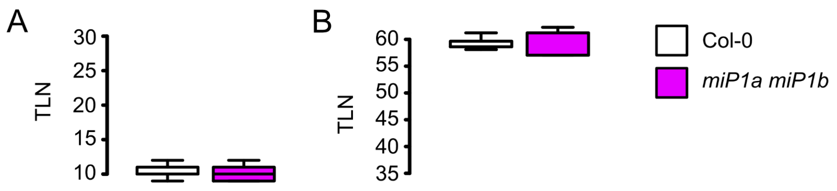

Supplementary Figure S5 – Flowering time analysis of *miP1a miP1b* mutants in different photoperiods.
(A) Analysis of flowering in a long-day regime (16 hours day - 8 hours night).
(B) Analysis of flowering in a short-day regime (8 hours day - 16 hours night).
Quantification of flowering by counting the total leaf number (TLN) at bolting. N=10. The center line of the box plots depicts the median and box limits indicate the 25th and 75th percentiles. The whiskers extend 1.5 times the interquartile range from the 25th and 75th percentiles.
